# Prognostic nutritional index before surgical treatment may serve as a prognostic biomarker for patients with upper tract urothelial carcinoma: A systematic review and meta-analysis

**DOI:** 10.3389/fnut.2022.972034

**Published:** 2022-09-23

**Authors:** Chunyang Meng, Lijian Gan, Kangsen Li, Fulin Yi, Lei Peng, Jinze Li, Yunxiang Li

**Affiliations:** ^1^Department of Urology, The Affiliated Nanchong Central Hospital of North Sichuan Medical College (University), Nanchong, China; ^2^Department of Anesthesiology, North Sichuan Medical College (University), Nanchong, China; ^3^Department of Urology, Institute of Urology, West China Hospital, Sichuan University, Chengdu, China

**Keywords:** prognostic nutritional index, upper tract urothelial carcinoma, prognostic biomarker, meta-analysis, PNI

## Abstract

**Objective:**

This meta-analysis aims to assess whether the prognostic nutritional index (PNI) score before treatment can be an independent biomarker of the prognosis of patients with upper tract urothelial carcinoma (UTUC).

**Materials and methods:**

We systematically search PubMed, Embase, Scopus database, and Cochrane Library, and the search time is up to April 2021. Use STATA 16.0 software for data processing and statistical analysis.

**Results:**

Six studies, including seven cohorts, were eventually included in our meta-analysis. The meta-analysis results showed that low PNI scores are associated with worse OS (HR: 1.92; 95% CI 1.60 to 2.30; *P* < 0.01), DFS/RFS/PFS (HR: 1.57; 95% CI 1.33 to 1.85; *P* < 0.01), and CSS/DSS (HR: 1.79; 95% CI 1.49 to 2.16; *P* < 0.01), which supported the PNI score as an independent prognostic biomarker for survival outcomes. The subgroup analysis and Begg’s test showed that the results were stable.

**Conclusion:**

Based on current evidence, this meta-analysis proves that the PNI score of UTUC patients before treatment is an independent prognostic biomarker. It performs well on OS, DFS/RFS/PFS, and CSS/DSS. This conclusion needs to be verified by a prospective cohort study with larger sample size and a more rigorous design.

**Systematic review registration:**

[https://www.crd.york.ac.uk/prospero/display_record.php?ID=CRD42022338503], identifier [CRD42022338503].

## Introduction

Upper tract urothelial carcinoma (UTUC) is a malignant tumor, that locates from the calyx system to the distal ureter. UTUC is relatively rare, accounting for only 5–10% of urothelial carcinoma (1, 2). Currently, the standard treatment of non-metastatic UTUC remains radical nephroureterectomy (RNU) with bladder cuff excision. However, approximately 60% of patients with UTUC are invasive at diagnosis, and the prognosis is poor (3). Previous studies show that the 5-year specific survival is < 50% for UTUC patients with pT2 or pT3 and < 10% for pT4 (2). Some preoperative and postoperative factors, such as tumor stage, tumor grade, tumor size, and lymph node involvement, were suggested to predict prognosis in UTUC (4). Nonetheless, not every UTUC patient can receive surgical treatment or undergo radical surgery (5). Thus, the potential pretreatment prognostic marker is particularly important in UTUC.

The prognostic nutritional index (PNI) was originally described by Onodera et al. (6), which were calculated by serum albumin levels and peripheral lymphocyte count (7). PNI is a simple and easily accessible index used to evaluate the perioperative immune and nutritional status and risk of post-operative complications (8). Research has shown that PNI has been validated as an independent prognostic factor for various types of cancer (8–10).

Although some studies have been published, the role of PNI as a predictor of prognosis is still controversial in UTUC (7, 11). This study aims to evaluate whether the PNI may serve as an independent prognostic biomarker for patients with upper tract urothelial carcinoma, to assist clinicians in improving the prognosis of UTUC patients.

## Materials and methods

### Literature search and eligibility criteria

Based on the guidelines of Preferred Reporting Items for Systematic Reviews (12), we performed a systematic search to identify studies in PubMed, Embase, Scopus database, and Cochrane Library. The latest search time was April 2022. Search terms included: “upper tract urothelial cancer,” “UTUC,” “malignant tumor,” “radical nephroureterectomy,” “treatment,” “surgical*,” “prognostic nutritional index,” “PNI,” “predict*,” “prognostic*,” “factor,” “indicators.” Combine the above search fields with logical operators to get as many search results as possible. Besides, some research references were searched manually.

The inclusion and exclusion of the study were as follows: (1) Upper tract urothelial cancer was pathologically diagnosed, and there were no other types of malignant or metastatic cancer. (2) Before treatment, the prognostic nutritional index was calculated. (3) All the patients received surgical intervention: NU or RNU and did not receive other surgical treatment during the same period. (4) The researchers followed up with the patients for a certain period and were able to obtain at least one of the over survival (OS), cancer-specific survival (CSS), disease-specific survival (DSS), recurrence-free survival (RFS), progression-free survival (PFS), or disease-free survival (DFS). (5) The effects between the low PNI group and the high PNI group on the prognosis of surgical patients were evaluated, and the hazard ratio (HR) was presented in the study. (6) The design type of included study was retrospective or prospective. Letters, case reports, reviews, repeated studies, studies unrelated to the topic, animal experiments, and research without available data were excluded.

The process of identifying studies was completed independently by two authors (CM and LG). At the same time, data extraction and quality assessment were performed for the included studies. Negotiating between the two authors resolved the differences, and a consensus result was reached.

### Quality evaluation

Based on the results of the identifying process, we used the NOS scale to assess the quality of included studies (13). The scale includes three question areas for selection, comparability, and exposure. The scale ranged from zero to nine stars, and studies with a score of six stars or more were considered high quality.

### Data extraction

The researchers used the standard table to extract the following information from included studies: first author’s name, publication year, region, study design, sample size, intervention, mean age, cutoff value, follow-up time, survival statistics, hazard ratio (HR) and 95% confidence intervals (95% CI).

### Data analysis

Data analysis was done by using Stata version 16.0 (StataCorp LP, University City, Texas, United States). Using the HR and its 95% CI of the multivariate analysis in each study to assess the importance of the PNI score for the prognosis of UTUC patients. In the meta-analysis, when the effect index is HR, the risk ratio is usually taken as the logarithm as the effect value (14). Therefore, we enter commands in the Stata 16 software to find the logarithmic values of HR, the upper limit of HR’s 95% CI, and the lower limit of HR’s 95% CI, and then perform the meta-analysis. The others can be extracted directly from the original study without conversion. We performed the *Q* test and χ^2^ test to value the heterogeneity between the included literatures. If *I*^2^ > 50%, the differences between the studies are considered significant, and random effect models are used (15). In addition, a sensitivity analysis is also carried out on this basis (16). We did subgroup analyses for each survival statistic based on the cutoff value. Begg’s test was used to test for publication bias between studies, and *P* < 0.05 was considered biased (17).

## Results

### Description of studies

**FIGURE 1:**
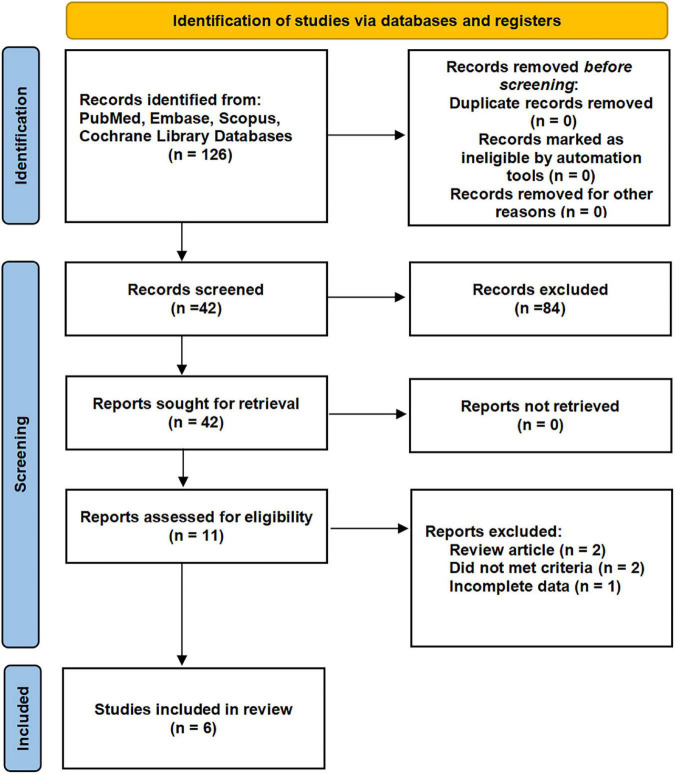
Flow diagram of the studies selection process.

By the search process, 214 studies were screened from the established database, and two studies were searched manually. Six studies, including seven cohorts, were eventually included in our meta-analysis (7, 11, 18–21). The detailed systematic search process is shown in Figure 1. The baseline data of the included studies are given in Table 1, including age, region, type of study design, sample size, surgical type, cutoff value, follow-up time, grouping, and survival outcomes. Six studies, including 2,324 patients, were published between 2015 and 2022. The included studies were all retrospective studies.

**TABLE 1:** Baseline data for studies included in the meta-analysis.

Author, year	Region	Study design	Sample size	Intervention	Age^a^	Cutoff^b^	Follow-up time^c^	Group by cutoff	Survival outcomes
Kim et al. (19)	Korea	Retrospective	277	NU^d^	63.7 (29.5–90)	45	57.2 mon (6.8–158.3)	PNI<45 (*n* = 44) PNI ≥ 45 (*n* = 233)	DSS^f^, DFS^g^
Huang et al. (20)	China	Retrospective	425	RNU^e^	65.9 ± 11.1	46.78	50 mon	PNI<46.78(*n* = 102) PNI ≥ 46.78(*n* = 323)	OS^h^, CSS^i^
Xue et al. (21)	China	Retrospective	717	RNU	67	46.91	38.5 mon (23–62)	PNI<46.91(*n* = 298) PNI ≥ 46.91(*n* = 419)	OS, CSS, RFS^j^
Itami et al. (18)	Japan	Retrospective	125	RNU	72 (38–90)	50	51 mon (6–227)	PNI ≤ 50 (*n* = 60) PNI>50 (*n* = 65)	OS, DSS
Zheng et al. (11)	China	Retrospective	253	RNU	67.59 ± 10.49	47.83	33.8 mon (16.7–64.4)	PNI<47.83(*n* = 100) PNI ≥ 47.83(*n* = 153)	OS, CSS, RFS
Zheng et al. (11)	China	Retrospective	272	RNU	65.87 ± 10.35	47.83	44.6 mon (26.8–65.3)	PNI<47.83(*n* = 146) PNI ≥ 47.83(*n* = 126)	OS, CSS, RFS
Liu et al.(7)	China	Retrospective	255	RNU	69 ± 10.37	50.5	43.93 mon (19.30–82.77)	PNI<50.5 (*n* = 174) PNI ≥ 50.5 (*n* = 81)	OS, CSS, PFS^k^

^a^Age, Mean ± SD/Mean(Range)/Mean. ^b^Cutoff, cutoff value of PNI score. ^c^Follow-up time, Mean/Mean(Range)/Mean[Interquartile range]. ^d^Nephroureterectomy. ^e^Radical nephroureterectomy. ^f^DSS, Disease-specific survival. ^g^DFS, Disease-free survival. ^h^OS, Over Survival. ^i^CSS, Cancer-specific survival. ^j^RFS, recurrence-free survival. ^k^PFS, progression-free survival.

### Quality assessment

According to the scoring rules of the NOS scale, we assessed the quality of the studies. The quality scores of the included studies are recorded in Table 2. The quality scores of all included studies are ≥ 6 stars and are considered high quality.

**TABLE 2:** Quality evaluation of the eligible studies with Newcastle–Ottawa scale.

Study	Selection				Comparability		Exposure			Total points
			
	REC	SNEC	AE	DO	SC	AF	AO	FU	AFU	
Kim et al. (19)	-	*	*	-	*	*	*	*	*	7
Huang et al. (20)	*	*	*	*	*	-	*	-	-	6
Xue et al. (21)	*	*	*	*	*	-	*	-	*	7
Itami et al. (18)	*	*	*	*	*	-	*	-	-	6
Zheng et al. (11)	*	*	*	*	*	-	*	-	*	7
Zheng et al. (11)	*	*	*	*	*	-	*	-	*	7
Liu et al. (7)	*	*	*	*	*	-	*	-	*	7

REC representativeness of the cohort, SNEC selection of the none posed cohort, AE ascertainment of exposure, DO demonstration that outcome of interest was not present at start of study, SC study controls most important factors, AF study controls for other important factors, AO assessment of outcome, FU follow-up long enough for outcomes to occur, AFU adequacy of follow-up of cohort (≥ 80%). *Indicates criterion met, -indicates significant of criterion not met.

### Survival outcomes

#### The relationship between over survival and prognostic nutritional index score

**FIGURE 2:**
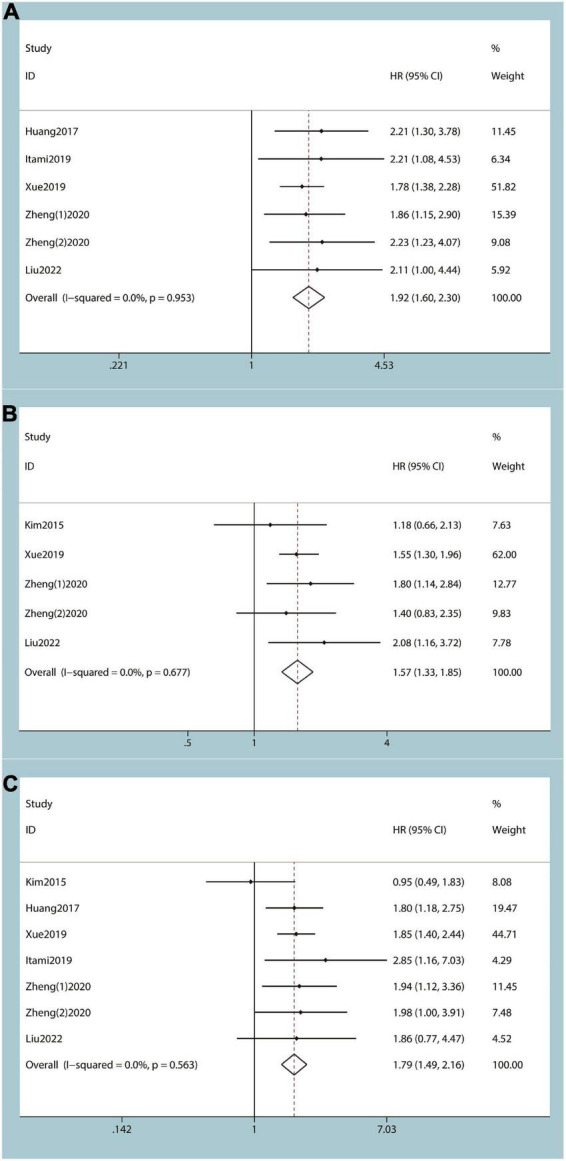
Forest plot and meta-analysis. **(A)** Forest plot and meta-analysis of the relationship between over survival (OS) and prognostic nutritional index (PNI) score. **(B)** Forest plot and meta-analysis of the relationship between disease-free survival/recurrence-free survival/progression-free survival, and prognostic nutritional index score. **(C)** Forest plot and meta-analysis of the relationship between cancer-specific survival/disease-specific survival, and prognostic nutritional index score.

Five studies, including six cohorts, revealed the correlation between preoperative PNI score and OS (7, 11, 18, 20, 21). According to the results of the heterogeneity test, there was no heterogeneity among the studies (*I*^2^ = 0%), and a fixed effects model was used to combine the effect size of each study. The outcomes of the meta-analysis demonstrated that lower preoperative PNI scores were associated with poorer OS (HR: 1.92; 95% CI 1.60 to 2.30; *P* < 0.01 Figure 2A).

#### The relationship between disease-free survival/recurrence-free survival/progression-free survival, and prognostic nutritional index score

A total of five eligible studies revealed the prognostic role of pre-treatment PNI score on DFS/RFS/PFS in patients with UTUC (7, 11, 19, 21). Since there was no heterogeneity among studies (*I*^2^ = 0%), we used a fixed effects model to perform the meta-analysis. The ultimate result showed that the lower the preoperative PNI score of UTUC patients, the decreased their DFS/RFS/PFS (HR: 1.57; 95% CI 1.33 to 1.85; *P* < 0.01 Figure 2B).

#### The relationship between cancer-specific survival/disease-specific survival, and prognostic nutritional index score

Six studies, including seven cohorts, showed the correlation between preoperative PNI score and CSS/DSS (7, 11, 18–21). Given the heterogeneity test outcome (*I*^2^ = 0%), we used the fixed effects model. Our results suggested that a lower level of preoperative PNI was associated with decreased CSS/DSS (HR: 1.79; 95% CI 1.49 to 2.16; *P* < 0.01 Figure 2C).

### Subgroup analysis

Owing to the lack of sufficient data, subgroup analysis was only performed in terms of cutoff value. Stratified analysis by the size of cutoff value also showed that a low pre-treatment PNI score was associated with the worse OS, DFS/RFS/PFS, and CSS/DSS (Table 3).

**TABLE 3:** Subgroup analysis of survival outcomes.

Subgroup	Cutoff value	Included cohort	Effect model	HR (95%CI)	*P*	Heterogeneity	
						
						*I*^2^(%)	P
**OS**							
Cut-off value	<47	2	fixed	1.85 (1.47, 2.32)	<0.01	0	0.464
	≥ 47	4	fixed	2.05 (1.52, 2.76)	<0.01	0	0.960
**CSS/DSS**							
Cut-off value	<47	3	fixed	1.70 (1.37, 2.12)	<0.01	0	0.176
	≥ 47	4	fixed	2.05 (1.44, 2.93)	<0.01	0	0.893
**RFS/DFS/PFS**							
Cut-off value	<47	2	fixed	1.51 (1.24, 1.83)	<0.01	0	0.392
	≥ 47	3	fixed	1.72 (1.28, 2.31)	<0.01	0	0.589

### Sensitivity analysis

**FIGURE 3:**
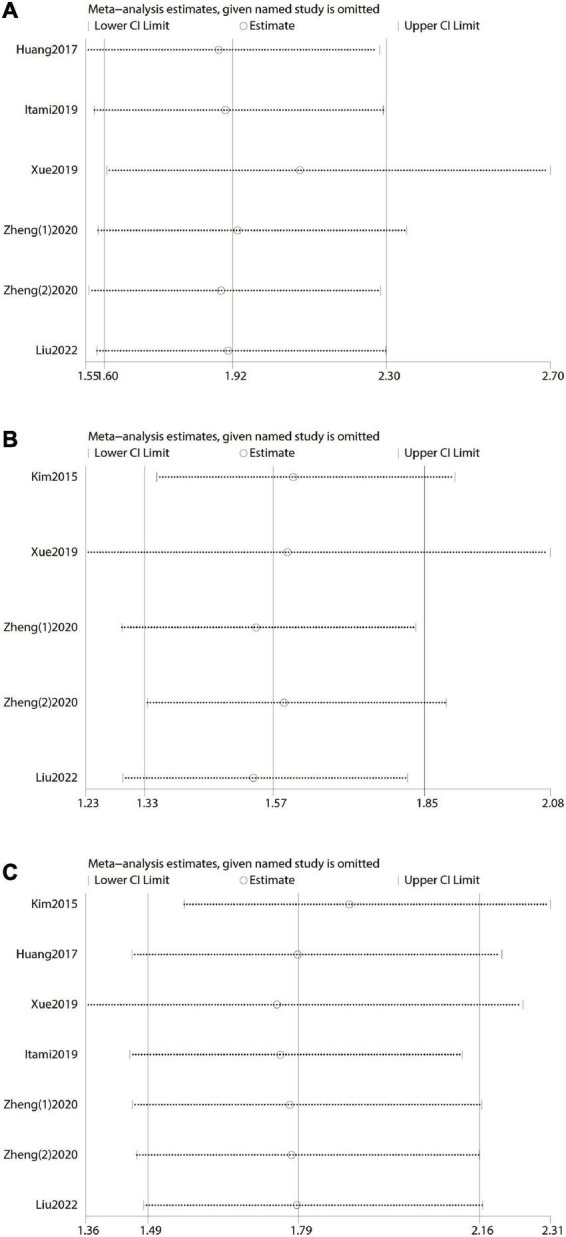
Forest plot and sensitivity analysis. **(A)** Forest plot and sensitivity analysis of the relationship between over survival (OS) and prognostic nutritional index (PNI) score. **(B)** Forest plot and sensitivity analysis of the relationship between disease-free survival/recurrence-free survival/progression-free survival, and prognostic nutritional index score. **(C)** Forest plot and sensitivity analysis of the relationship between cancer-specific survival/disease-specific survival, and prognostic nutritional index score.

Sensitivity analysis was performed by excluding one single study once a time and recalculating the effect size of the remaining part. It reflected the impact of the individual on the whole. The result of our sensitivity analysis showed that no single study significantly influenced the pooled HR and 95% CI. This meant that our results were stable (Figure 3).

### Publication bias

**FIGURE 4:**
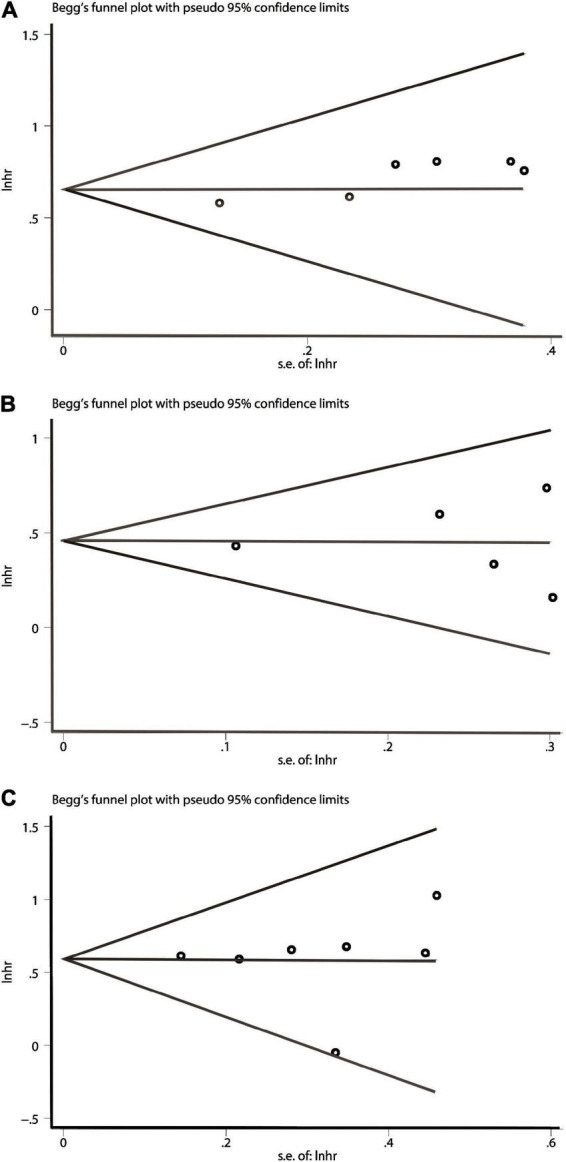
Begg’s test for publication bias. **(A)** Over survival (OS). **(B)** Disease-free survival/recurrence-free survival/progression-free survival (DFS/RFS/PFS). **(C)** cancer-specific survival/disease-specific survival (CSS/DSS).

In terms of OS or DFS/RFS/PFS or CSS/DSS, Publication bias was evaluated by Begg’s test. The *P* values of them were all above 0.05, showing no significant publication bias was found (Figure 4). That is to say, the results of our meta-analysis were reliable based on the available articles.

## Discussion

Although RNU was the standard treatment for UTUC, approximately one-third of UTUC patients who undergo surgery will experience early recurrence, and 80% of them will eventually die from UTUC (22). The current pre-operative prognostic indicters, such as c-reactive protein (23), fibrinogen (24), pre-treatment lymphocyte-monocyte ratio (25), and pre-treatment neutrophil-to-lymphocyte ratio (26), are helpful to the prediction of survival outcomes of UTUC patients, but it only focuses on inflammatory conditions. As is well-known, the nutritional status of tumor patients is closely related to their prognosis (27). Based on body mass index, serum albumin, and preoperative weight loss, Gregg et al. developed a simple model to predict 90-day mortality and 3-year OS in patients with bladder cancer (28). Moreover, a study conducted by Huang et al. (29) showed that decreased preoperative pre-albumin levels as an independent prognostic factor for CSS and OS in patients with UTUC.

Prognostic nutritional index (PNI) was a simple and accessible preoperative indicator that could provide a comprehensive and objective assessment of the inpatient’s condition. Due to the particularity of the PNI score composition, it could reflect the body’s protein metabolism and immune function, which were usually associated with the body’s nutritional status and immune response. Several retrospective studies have reported that PNI may be one of the potential predictors of postoperative survival outcomes in UTUC patients (7, 18). Consequently, we performed a meta-analysis to evaluate the impact of PNI on the prognosis outcomes in UTUC patients after surgical treatment.

This meta-analysis provided an evidence-based medicine analysis of six published studies exploring the prognostic and survival indicators of PNI in patients with UTUC. Our results showed that low PNI scores are associated with worse OS, DFS/RFS/PFS, and CSS/DSS, which supported the PNI score as an independent prognostic biomarker for survival outcomes.

Increasing evidence shows that the presence of nutritional deficiencies and systematic inflammatory response might play an important position in the development and progress of human cancers (30). Albumin is the main component of serum proteins, reflecting the nutritional status of the human body to a certain extent. It could regulate inflammatory reaction and exert antioxidant effects against carcinogens (31). In addition, low albumin levels reflect nutritional deficiencies, which could lead to reduced immune function and poor anticancer response (32). Recently, studies have shown that preoperative low albumin is an independent predictor of poor prognosis in patients with malignant tumors (33, 34). A study involving 214 glioblastoma patients have shown that serum albumin levels correlated with OS (HR = 0.966; 95% CI 0.938 to 0.995, *P* = 0.023) (35). Another study indicated that compared with those with hypoalbuminemia, vulvar cancer patients with normal albumin levels had a longer 5-year OS (58.6 vs. 17.1%, *P* = 0.004) (36). Furthermore, albumin levels are related to the systemic inflammatory response (37). Previous studies have found that albumin synthesis was reduced with the release of tumor necrosis factor. Under inflammatory conditions, the increased permeability of the vascular endothelium leads to albumin escape (38). Ishizuka et al. found that the relationship between hypoalbuminemia and poor postoperative outcome in patients with colorectal cancer was associated with increased inflammation (39). These studies proved the vital role of serum albumin as a nutritional indicator in cancer and inflammation, which supported the conclusions of this meta-analysis.

The relationship between inflammation and cancer was first described in the mid-19th century (40). In recent years, there has been increasing evidence of an association between inflammation, which is thought to be a pivotal event in the early development of cancer, and poor oncological prognosis (41, 42). Lymphocytes are common inflammatory cells in the tumor microenvironment and play an important anti-tumor effect in the immune system (42). In the advanced stage, tumor cells could destroy lymphocytes by editing proapoptotic ligands, and eventually achieve immune escape. In addition, the anti-tumor immune response mediated by CD8^+^ T lymphocytes also has an important role in the treatment of tumors. However, it doesn’t work endlessly. Some cancer-associated cells, such as fibroblasts, macrophages, and regulatory T cells, might produce an immune barrier to counteract the immune function of T cells, leading to a decrease in the number of T lymphocytes, tumor cell proliferation, and metastasis (43).

To our knowledge, this is the first meta-analysis to focus on the prognostic value of PNI in UTUC patients, and we followed PRISM guidelines strictly to perform this meta-analysis. However, some limitations cannot be avoided. First, the included studies are all retrospective studies, and the level of evidence is low. Second, the included studies are limited to East Asia, making the research results less universal. Third, due to the small number of studies available, not enough information is available to perform subgroup analysis to identify high-risk populations.

## Conclusion

In conclusion, this meta-analysis revealed that the preoperative PNI is a potential independent biomarker of the postoperative prognosis of UTUC patients. A low PNI score predicts worse OS, DFS/RFS/PFS, and CSS/DSS in patients. Therefore, the clinician can individualize disease management for patients based on the PNI score for better treatment outcomes. This conclusion requires a larger sample size and a more rigorously designed prospective study to prove it.

## Data availability statement

The original contributions presented in this study are included in the article/Supplementary material, further inquiries can be directed to the corresponding author.

## Author contributions

YL conceived and designed the experiments. CM, LP, and KL analyzed the data. LG, KL, and JL contributed reagents, materials, and analysis. CM, LG, and FY wrote the manuscript. All authors have read and approved the final manuscript.
